# A Low-Grade Fibromyxoid Sarcoma of the Internal Abdominal Oblique Muscle

**DOI:** 10.1155/2016/8524030

**Published:** 2016-05-10

**Authors:** Masakazu Hashimoto, Kei Koide, Michinori Arita, Koji Kawaguchi, Yoshihiro Mikuriya, Jun Iwata, Toshiyuki Iwamoto

**Affiliations:** ^1^Department of Surgery, Chuden Hospital, 3-4-27 Otemachi, Naka-Ku, Hiroshima 730-8562, Japan; ^2^Department of Diagnostic Pathology, Kochi Health Sciences Center, 2125-1 Ike, Kochi 781-8555, Japan; ^3^Department of Pathology, Chuden Hospital, 3-4-27 Otemachi, Naka-Ku, Hiroshima 730-8562, Japan

## Abstract

A low-grade fibromyxoid sarcoma (LGFMS) is a rare tumor, with a benign histologic appearance but malignant behavior. This report describes a 74-year-old man with an internal abdominal oblique muscle mass. The tumor appeared as a well-defined ovoid mass on computed tomography, with mild uptake on fluorine-18-fluorodeoxyglucose positron-emission tomography images. Radical resection with wide safe margins was performed. Histologically, the tumor was composed of spindle-shaped cells in a whorled growth pattern, with alternating fibrous and myxoid stroma. MUC4 expression, a highly sensitive and specific immunohistochemical marker for LGFMS, was detected. Therefore, we diagnosed the tumor as LGFMS. At the 3-month follow-up, there was no sign of recurrence or metastasis. We report the first case of LGFMS arising from the internal abdominal oblique muscle.

## 1. Background

Soft tissue tumors are uncommon tumors accounting for only approximately 1% of cancers in adults, and it is often difficult to diagnose these tumors [1]. Owing to the small numbers of these neoplasms, it is difficult to perform systematic research and to develop optimal approaches for treatment and diagnosis of these patients. Unfortunately, many patients still undergo improper initial diagnosis and treatment.

A low-grade fibromyxoid sarcoma (LGFMS) is a rare variant of the spindle cell tumor that is composed of collagen-rich and myxoid parts [2]. Owing to its variable morphology, LGFMS can be difficult to distinguish from benign mesenchymal tumors and other low-grade sarcomas. Clinically, LGFMS develop mainly in the subcutaneous or superficial soft tissue overlying the muscles of the trunk or proximal four limbs in middle-aged adults. LGFMS sometimes recurs locally and distantly [3]. Recently, immunohistochemistry has been playing a key role in the diagnosis of LGFMS. It is identified by using MUC4 staining, which can be helpful to distinguish this tumor type from histologic mimics [4].

This report describes a 74-year-old man with LGFMS of the right internal abdominal oblique muscle.

## 2. Case Presentation

**Figure 1:**
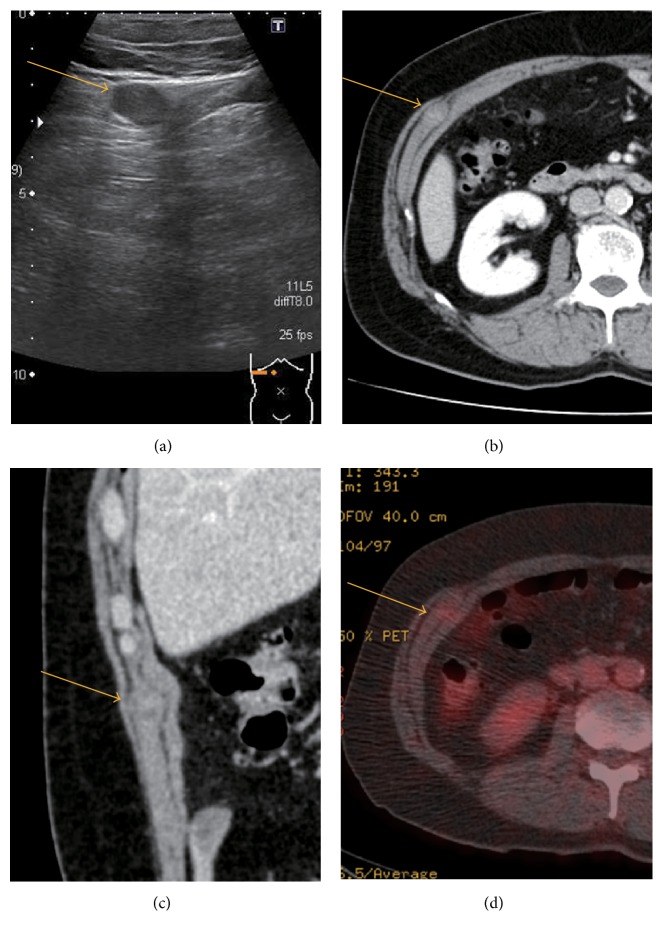
(a) US shows a well-demarcated hypoechoic mass in the abdomen. (b) Axial view and (c) coronal view of the CT abdomen with intravenous contrast. The mass shows a heterogeneous density in the internal abdominal oblique muscle. (d) FDG-PET demonstrated low FDG uptake in the tumor. The maximum SUV of the tumor was 1.4.

The patient was a 74-year-old man who had an abdominal wall mass identified on abdominal ultrasonography during a routine examination (Figure 1(a)). There was no previous history of a significant injury to his abdomen. On physical examination, the elastic firm mass measured approximately 20 × 20 mm without tenderness. Computed tomography (CT) revealed a low-density mass in the right internal abdominal oblique muscle (Figure 1(b)). On contrast-enhanced CT, the mass was mildly enhanced nonhomogeneously (Figure 1(c)). The mass was not detectable on a CT image acquired 5 years previously. Fluorine-18-fluorodeoxyglucose (FDG) positron-emission tomography (PET) imaging demonstrated low FDG uptake in the mass in the right internal abdominal oblique muscle. The maximum standardized uptake value (SUV-max) of the tumor was 1.4 (Figure 1(d)). FDG-PET imaging did not reveal any other distant metastases.

**Figure 2:**
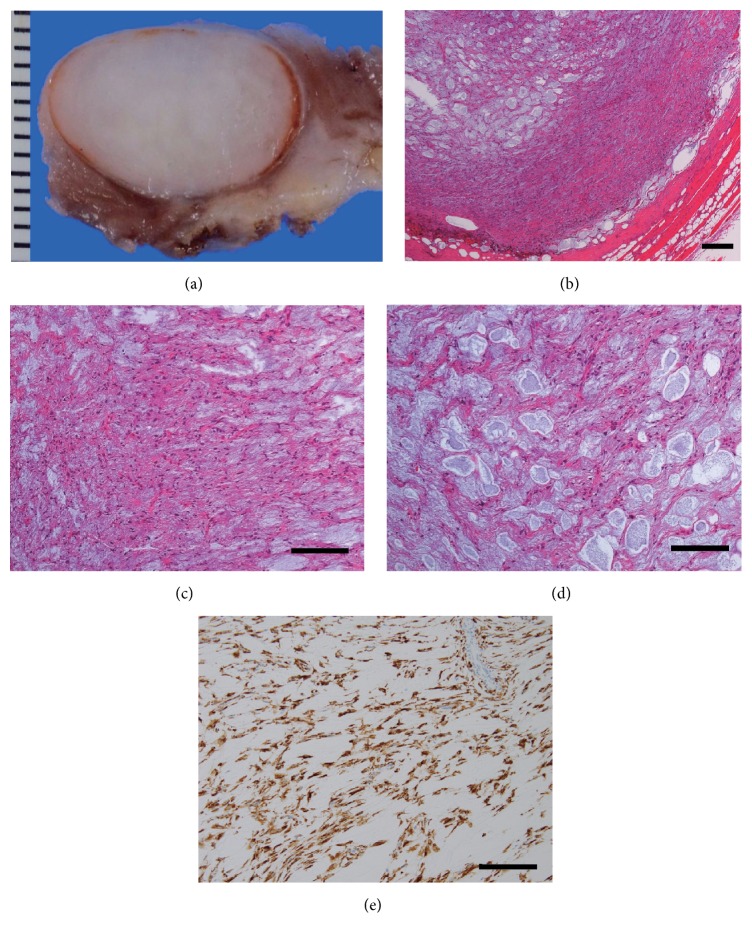
(a) The cut surface of the tumor revealed that the lesion was pale white and glistening in appearance. (b) A microscopic examination showed alternating areas with a fibrous and myxoid stroma. Scale bar is 500 *μ*m. (c) Low-power view of the myxoid zone. Scale bar is 50 *μ*m. (d) Low-power view of the fibrous zone. Scale bar is 50 *μ*m. (e) The tumor shows diffuse cytoplasmic expression of MUC4, characteristic of LGFMS.

For diagnosis and treatment, en bloc resection of the tumor was performed via wide resection. The resected specimen contained a mass with a pseudocapsule. On gross examination, the cut surface of the tumor revealed that the lesion was pale white and glistening (Figure 2(a)).

Histopathological examination demonstrated that the tumor was contained within a thin fibrous capsule and was well demarcated from the surrounding muscle and soft tissue. The tumor cells were spindle-cell-shaped fibroblast-like cells within whirling collagenous stroma. There were sporadic myxoid areas within the whirling collagenous stroma. There were sporadic areas of increased cellularity, and the tumor cells were occasionally multinucleated or stellate in shape. The nuclei of the tumor cells were mildly pleomorphic and hyperchromatic, but these features were not diagnostic for unequivocal malignancy (Figures 2(b) and 2(c)). On immunohistochemical examination, the tumor cells were negative for desmin, S100, smooth muscle actin, CD34, and CD117 and were positive for MUC4 (Figure 2(d)). The tumor was diagnosed as LGFMS.

The patient had not experienced either local recurrence or distant metastasis at the final follow-up 3 months after surgery.

## 3. Discussion

LGFMS occurs most commonly in the deep soft tissues of the proximal extremities and trunk. Other sites include the chest wall, hip, inguinal region, axilla, retroperitoneum, mesentery, pelvis, and maxilla [5–8]. To our knowledge, this is the first report of an LGFMS occurring on the internal abdominal oblique muscle.

There are a few reports of LGFMS that were positive on FDG-PET. SUVs of the masses ranged from 1.8 to 4.0 [9–11]. The SUV-max in our case was 1.4, and the size of the tumor in our case was smaller than the previously reported cases. Williams et al. [9] reported that FDG-PET could be useful to demonstrate sites of possible metastasis and direct biopsy for rare soft tissue sarcomas but is of uncertain negative predictive value for small tumors. Maretty et al. [12] claimed that small tumors were PET negative on the initial scan, so they were not removed until 3 months later, after metastases were observed. They concluded that PET-CT should be used with caution in patients with LGFMS.

In our case, on microscopic examination, the tumor showed alternating fibrous and myxoid areas and had spindle and asteroid fibroblast-like tumor cells in this myxoid background, evident on hematoxylin and eosin staining (H&E). Although the tumor cell nuclei were mildly pleomorphic and hyperchromatic, the malignant nature of the tumor was not readily discernible. Our first differential diagnoses were “intramuscular myxoma,” “nodular fasciitis,” and “cellular myxoma,” but none of them fit the histological features of the tumor. However, on immunohistochemical analysis, the tumor was unexpectedly positive for MUC4, but negative for desmin, S100, smooth muscle actin, CD34, and CD117. Our diagnostic possibility was narrowed to “LGFMS.” Doyle et al. reported that MUC4 was a highly sensitive and specific immunohistochemical marker for LGFMS [4]. They reported that all 49 LGFMS cases (100%) showed cytoplasmic staining for MUC4 and all other tumor types were negative for MUC4, other than 6 (30%) monophasic synovial sarcomas. Among other soft tissue tumors, MUC4 is a sensitive and useful marker for identifying only sclerosing epithelioid fibrosarcoma, which has similarities to LGFMS [13]. When we reviewed the H&E slides of the tumor, we found the features to be consistent with LGFMS.

Although patients with LGFMS are often misdiagnosed with benign tumors such as fibromatosis and neurofibroma instead of LGFMS, adequate surgical excision of the tumor is undoubtedly necessary because of the frequent recurrence of LGFMS. The local recurrence rate proved to be clearly lower in specimens with adequate margins [3, 5]. Recent study reports that the rate of metastases in LGFMS is 45% [3], although earlier studies claimed it rarely had metastatic potential. The treatment of metastatic LGFMS is difficult and may include multiagent chemotherapy and repeated and selective surgery of operable metastases [12]. Moreover, Evans reported that clinical and histological responses were quite poor for distant metastases and nonresectable lesions [3]. In our case, the resected specimen had a wide margin and the tumor was small. The patient was followed-up with clinical physical examinations and CT every 3 months.

## 4. Conclusion

In conclusion, this is a case of LGFMS that formed in the internal abdominal oblique muscle of a 74-year-old man. Although LGFMS can be difficult to distinguish from a benign tumor on clinical examination, it should be correctly diagnosed on histological and immunohistochemical examinations in order to ensure adequate treatment.

## Abbreviations

LGFMS: Low-grade fibromyxoid sarcoma
FDG-PET: Fluorine-18-fluorodeoxyglucose positron-emission tomography
CT: Computed tomography.

## References

[1] Thway K. (2009). Pathology of soft tissue sarcomas. *Clinical Oncology*.

[2] Evans H. L. (1987). Low-grade fibromyxoid sarcoma. A report of two metastasizing neoplasms having a deceptively benign appearance. *American Journal of Clinical Pathology*.

[3] Evans H. L. (2011). Low-grade fibromyxoid sarcoma: a clinicopathologic study of 33 cases with long-term follow-up. *The American Journal of Surgical Pathology*.

[4] Doyle L. A., Möller E., Cin P. D., Fletcher C. D. M., Mertens F., Hornick J. L. (2011). MUC4 is a highly sensitive and specific marker for low-grade fibromyxoid sarcoma. *American Journal of Surgical Pathology*.

[5] Goodlbd J. R., Mentzel T., Fletcher C. D. M. (1995). Low grade fibromyxoid sarcoma: clinicopathological analysis of eleven new cases in support of a distinct entity. *Histopathology*.

[6] Takanami I., Takeuchi K., Naruke M. (1999). Low-grade fibromyxoid sarcoma arising in the mediastinum. *Journal of Thoracic and Cardiovascular Surgery*.

[7] Alevizopoulos A., Mygdalis V., Tyritzis S., Stravodimos K., Constantinides C. A. (2012). Low-grade fibromyxoid sarcoma of the renal pelvis: first report. *Case Reports in Nephrology and Urology*.

[8] Spalthoff S., Bredt M., Gellrich N., Jehn P. (2016). A rare pathology: low-grade fibromyxoid sarcoma of the maxilla. *Journal of Oral and Maxillofacial Surgery*.

[9] Williams H. T., Gossage J. R., Allred T. J., Kallab A. M., Pancholy A., Anstadt M. P. (2004). F-18 FDG positron emission tomography imaging of rare soft tissue sarcomas: low-grade fibromyxoid sarcoma and malignant hemangiopericytoma. *Clinical Nuclear Medicine*.

[10] Yamashita H., Endo K., Takeda C., Teshima R., Osaki M., Yoshida H. (2013). Intramuscular myxoma of the buttock mimicking low-grade fibromyxoid sarcoma: diagnostic usefulness of MUC4 expression. *Skeletal Radiology*.

[11] Konecna J., Liberale G., Haddad J., De Saint-Aubain N., El Nakadi I. (2015). Diffuse intra-abdominal low grade fibromyxoid sarcoma with hepatic metastases: case report and review of the literature. *International Journal of Surgery Case Reports*.

[12] Maretty-Nielsen K., Baerentzen S., Keller J., Dyrop H. B., Safwat A. (2013). Low-grade fibromyxoid sarcoma: incidence, treatment strategy of metastases, and clinical significance of the FUS gene. *Sarcoma*.

[13] Doyle L. A., Wang W.-L., Dal Cin P. (2012). MUC4 is a sensitive and extremely useful marker for sclerosing epithelioid fibrosarcoma: association with FUS gene rearrangement. *The American Journal of Surgical Pathology*.

